# Adaptation of the Invasive Plant *Sphagneticola trilobata* to Flooding Stress by Hybridization with Native Relatives

**DOI:** 10.3390/ijms25126738

**Published:** 2024-06-19

**Authors:** Qilei Zhang, Guangxin Chen, Weiqian Ke, Changlian Peng

**Affiliations:** 1 Guangzhou Key Laboratory of Subtropical Biodiversity and Biomonitoring, Guangdong Provincial Key Laboratory of Biotechnology for Plant Development, School of Life Sciences, South China Normal University, Guangzhou 510631, China; dalei45666@163.com (Q.Z.); gxchen2019@126.com (G.C.); keweiqscnu@163.com (W.K.); 2 Research Institute of Tropical Forestry, Chinese Academy of Forestry, Guangzhou 510520, China

**Keywords:** biological invasion, hybridization, flooding, *Sphagneticola trilobata*

## Abstract

Hybridization is common between invasive and native species and may produce more adaptive hybrids. The hybrid (*Sphagneticola* × *guangdongensis*) of *Sphagneticola trilobata* (an invasive species) and *S. calendulacea* (a native species) was found in South China. In this study, *S. trilobata*, *S. calendulacea*, and *Sphagneticola* × *guangdongensis* were used as research materials to explore their adaptability to flooding stress. Under flooding stress, the ethylene content and the expression of key enzyme genes related to ethylene synthesis in *Sphagneticola* × *guangdongensis* and *S. calendulacea* were significantly higher than those in *S. trilobata*. A large number of adventitious roots and aerenchyma were generated in *Sphagneticola* × *guangdongensis* and *S. calendulacea*. The contents of reactive oxygen species and malondialdehyde in *Sphagneticola* × *guangdongensis* and *S. calendulacea* were lower than those in *S. trilobata*, and the leaves of *S. trilobata* were the most severely damaged under flooding stress. The results indicate that hybridization catalyzed the tolerance of *Sphagneticola* × *guangdongensis* to flooding stress, and the responses of *Sphagneticola* × *guangdongensis* to flooding stress were more similar to that of its native parent. This suggests that hybridization with native relatives is an important way for invasive species to overcome environmental pressure and achieve invasion.

## 1. Introduction

Biological invasions threaten ecosystem function and biological diversity and have long-term ecological consequences [1]. At the same time, it is common for invasive species to hybridize with native species during biological invasion, which may produce more adaptive hybrids [2,3,4]. For example, *Spartina angelica* is a hybrid species produced by natural hybridization between *S*. *altermiflora* and *S*. *marime*, and its invasive ability is stronger than that of its parents [4,5]. Hybridization is a mechanism of invasive evolution in exotic plant species that can generate new hybrids that spread wider than their parents. For example, the invasive species *Helianthus paradoxes* is a hybrid produced by natural hybridization between *H*. *annuus* and *H*. *petiolaris* [6]. Hybridization is particularly important under constantly changing environmental conditions [7]. Heterosis is the result of the non-additive phenotypic expression of one or more traits, which may be regulated by epigenetics and genetics. Under environmental pressure, heterosis may lead to hybrid varieties with extreme phenotypes, exhibiting higher adaptability than their parents [8].

With global warming, frequent outbreaks of extreme weather, such as heavy rainfall and floods, have led to long-term flooding in some areas [9,10]. Water is one of the essential environmental factors for plants, but flooding is detrimental to the growth and development of terrestrial plants. Its distribution in the environment regulates the distribution, diversity, and succession characteristics of vegetation [11].

Most terrestrial plants are susceptible to damage under flooding events. The damage caused by flooding to plants is mainly due to a significant decrease in the diffusion rate of gases in water. Thus, the gas exchange between the submerged plant tissue and the environment is hindered. This affects the availability of CO_2_ and O_2_, reduces the production of photosynthetic products, and ultimately leads to plant mortality [12,13]. In plants, floods affect the stomatal conductance, leading to a decrease in the net photosynthetic rate (Pn) of leaves. The decrease in the net photosynthetic rate may be due to the decrease in the CO_2_ concentration caused by changes in the stomatal aperture [14,15]. The decrease in the Pn alters the flow of electrons along the electron transport chain, leading to the production of more reactive oxygen species (ROS). Proteins, lipids, and nucleic acids will be destroyed due to the increase in ROS activity, leading to further reductions in the Pn [16].

The roots of plants are the most susceptible organ to flooding. After the soil is covered by floods, the oxygen content in it decreases, causing the plant roots to be in an anaerobic environment [15]. Under hypoxic conditions, the absorption rate of minerals by the root system decreases, which cannot meet the growth needs of the aboveground parts of plants, resulting in slow plant growth [17]. Flooding not only alters soil compaction and microbial composition around roots but also intensifies competition among root microorganisms for nutrients and oxygen [18]. Root hypoxia causes energy imbalance, excessive production of ROS, and nitric oxide in the root system, thereby damaging the root tissue [19]. Therefore, the damage of flooding to plants is multifaceted and can damage the normal development and function of root systems. Ultimately, this leads to the loss of mineral absorption capacity in the root system, affecting the growth of the entire plant [20,21].

Plants have developed adaptive strategies in flooded environments, which have improved their tolerance to flooding stress. Stimulating growth to escape flooded conditions is one strategy; in order to obtain sufficient carbon dioxide, light, and oxygen, the upper stems and leaves of plants are exposed to the water surface [22]. Flood-tolerant plants have adopted a strategy of hypoxia quiescence syndrome, which extends their survival time in water by maintaining lower energy and carbon consumption [23]. Faced with flooding, adaptive changes occur in the roots and stems of plants, thereby improving their tolerance to flooding. For example, the formation of aerenchyma in roots and stems facilitates gas flow, accelerates stem elongation, and promotes adventitious root growth [24,25]. Flooding stress stimulates the synthesis and accumulation of ethylene, leading to the formation of aerenchyma, and interconnecting plant bodies form a unified entity. In addition, ethylene also promotes the formation of adventitious roots [26,27], which replaces the primary roots that die from hypoxia and obtains more nutrients and oxygen. Additionally, the antioxidant capacity is enhanced under flooding stress to remove excess ROS, which reduces damage to plants [28].

*Sphagneticola trilobata* (L.) Pruski is an invasive plant species in South China that originated in South and Central America. A hybrid, *Sphagneticola* × *guangdongensis* (Asteraceae, Heliantheae), found in South China, is the hybrid of *S*. *trilobata* and its native relative *S*. *calendulacea* (L.) Pruski [29,30]. The growth potential of the hybrid species *Sphagneticola* × *guangdongensis* is higher than that of the native species *S. calendulacea* and comparable to the invasive species *S. trilobata*, and its light energy utilization efficiency is higher than that of both the invasive and native species [29]. Under N deposition, the competitiveness of *Sphagneticola* × *guangdongensis* is similar to that of the invasive species and more sensitive [31]. We previously reported that the tolerance of *Sphagneticola* × *guangdongensis* to cadmium was stronger than its parents [32,33], and its tolerance to drought stress was higher than the native species and lower than the invasive species [34,35]. However, the tolerance level of *Sphagneticola* × *guangdongensis* to flooding stress is largely unknown. The aim of this study was to investigate the tolerance of the hybrid species *Sphagneticola* × *guangdongensis* to flooding stress and explore whether the invasive species *S. trilobata* could improve its adaptability to flooding stress by hybridizing with the native species *S. calendulacea*.

## 2. Results

### 2.1. Phenotypic Characteristics

**Figure 1:**
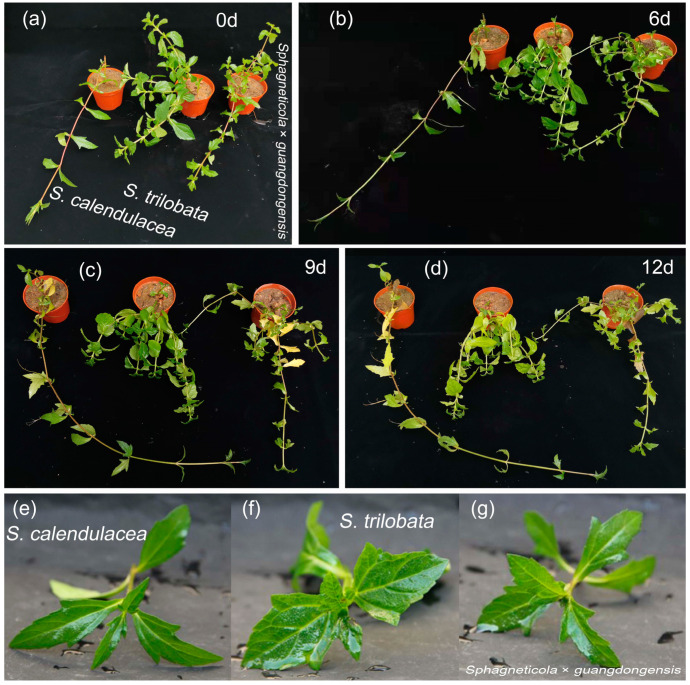
Phenotypic changes in the hybrid species *Sphagneticola* × *guangdongensis*, invasive species *S. trilobata,* and native species *S. calendulacea* at 0, 6, 9, and 12 days under flooding stress (**a**–**d**); the phenotypic changes in *Sphagneticola* × *guangdongensis* and its parents after 2 days of flooding stress (**e**–**g**).

By observing the phenotypic changes in *Sphagneticola* × *guangdongensis* and its parents under water flooding stress (Figure 1), it was found that the leaves of both *Sphagneticola* × *guangdongensis* and its two parents exhibited decay or shedding with prolonged flooding. However, the effects of flooding stress on the three species were different, and the invasive species *S. trilobata* suffered the most severe damage. After 2 days of flooding stress, necrotic spots were observed on the young leaves of the invasive species *S. trilobata* (Figure 1f). However, the young leaves of the hybrid species *Sphagneticola* × *guangdongensis* and the native species did not show necrotic spots (Figure 1e,g).

### 2.2. Accumulation of ROS and Changes in Photosynthetic Capacity

**Figure 2:**
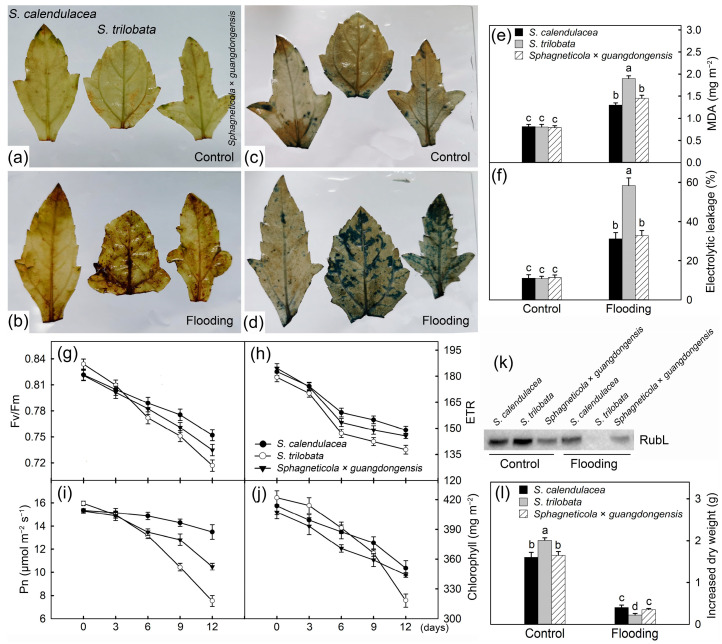
Tissue localization of hydrogen peroxide (**a**,**b**) and superoxide anions (**c**,**d**); changes in malondialdehyde (MDA, **e**) content, electrolytic leakage (**f**), Rubisco large subunit (RubL, **k**), and increased dry weight (**l**) under flooding stress for 12 days, compared with the control. Changes in the maximum photochemical efficiency (Fv/Fm, **g**), photosynthetic electron transport rate (ETR, **h**), net photosynthetic rate (Pn, **i**), and content of chlorophyll (**j**) under flooding stress. There were five biological replicates. Different letters (a, b, c, d) above bars indicate significant differences (*p* < 0.05).

Flooding stress led to membrane lipid peroxidation, a large amount of production and accumulation of ROS, and an increase in electrolyte leakage rates. H_2_O_2_ was detected with DAB staining, and O_2_^–^ was detected with NBT staining at 12 days after flooding stress. The highest levels of H_2_O_2_ and O_2_^–^ were detected in the leaves of the invasive species *S. trilobata* (Figure 2b,d). The degree of lipid peroxidation in cell membranes can be reflected by their MDA content. Flooding stress caused a significant increase in the MDA content in the leaves (F = 41.68; df = 5, 24). As shown in Figure 2e, the content of MDA in the invasive species *S. trilobata* was the highest, while it was lower in *Sphagneticola* × *guangdongensis* and *S. calendulacea*. The MDA content in the invasive species was significantly higher than that in *Sphagneticola* × *guangdongensis* (*p* < 0.01) and the native species (*p* < 0.01) but not significantly different between *Sphagneticola* × *guangdongensis* and the native species (*p* = 0.74). *Sphagneticola* × *guangdongensis* and *S. calendulacea* showed lower electrolytic leakage than *S. trilobata* under flooding stress (Figure 2f) (F = 93.98; df = 5, 24).

During the flooding, the Fv/Fm and ETR values in the leaves of *Sphagneticola* × *guangdongensis* and its parents gradually decreased (Figure 2g,h). After 6 days of flooding, the Fv/Fm and ETR values were the lowest in the leaves of the invasive species (*S. trilobata*), second lowest in the hybrid species *Sphagneticola* × *guangdongensis*, and highest in the native species *S. calendulacea*. The Pn value gradually decreased under flooding stress. The Pn was higher in *Sphagneticola* × *guangdongensis* than in *S. trilobata* after 9 d of flooding (Figure 2i).

The contents of chlorophyll and Rubisco are closely related to the photosynthesis of plant leaves, and they decreased after 12 days of flooding stress (Figure 2j), which is consistent with the results of the Pn. Western blotting analysis was carried out on a Rubisco large subunit (RubL) (Figure 2k), and it could not be detected in the invasive species *S. trilobata*. Compared to the control, flooding stress significantly reduced the increase in biomass (F = 470.82; df = 5, 24; *p* < 0.05). Under flooding stress, the increase in the biomass of *S. trilobata* was significantly lower than that of *Sphagneticola* × *guangdongensis* (*p* = 0.014) and *S. calendulacea* (*p* = 0.002). There was no significant difference in the biomass changes between *Sphagneticola* × *guangdongensis* and *S. calendulacea* (*p* = 0.406). However, without flooding stress, the magnitude of the biomass increase was significantly larger in the invasive species, compared to that of the *Sphagneticola* × *guangdongensis* and the native species (*p* < 0.01). These results imply that the invasive species *S. trilobata* suffered the most severe damage under flooding stress (Figure 2l).

### 2.3. Adventitious Roots and Aerenchyma

**Figure 3:**
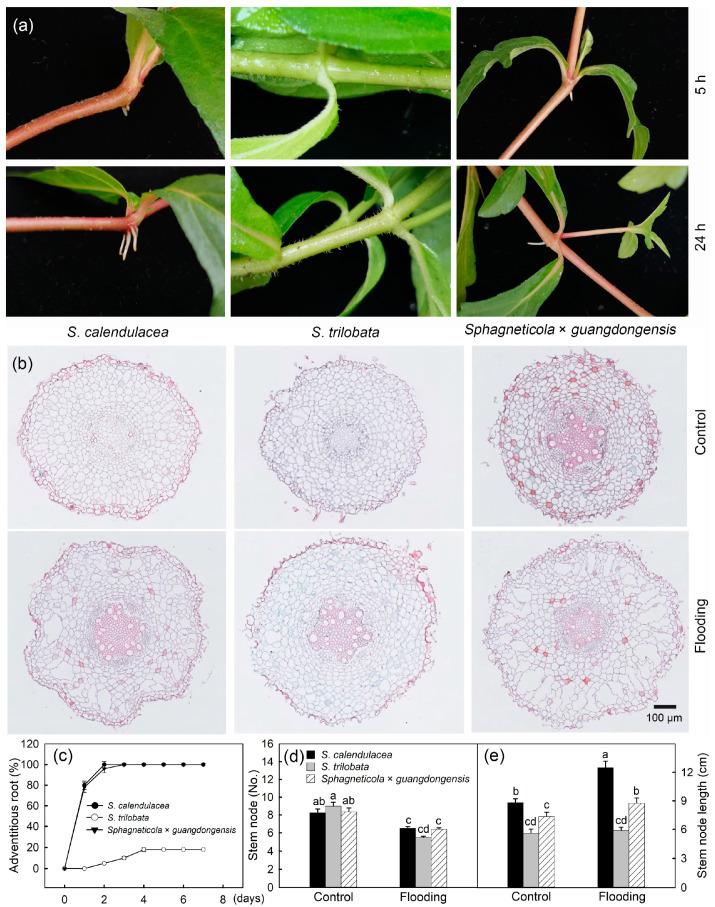
Adventitious roots under flooding stress for 5 and 24 h (**a**). The occurrence rate of adventitious roots under flooding stress (**c**). The biological replicates were forty. Aerenchyma (**b**), number of stem nodes (**d**), and length of stem nodes (**e**) under flooding stress for 12 days, compared with the control. There were ten biological replicates. Different letters (a, b, c, d) above bars indicate significant differences (*p* < 0.05).

Adventitious roots were observed in the hybrid species *Sphagneticola* × *guangdongensis* and the native species *S. calendulacea* under flooding stress for 5 h and 24 h but did not appear in the invasive species *S. trilobata* (Figure 3a). After 12 days of flooding, there were differences in the aerenchyma, with a large amount of aerenchyma present in *Sphagneticola* × *guangdongensis* and the native species (Figure 3b). The results showed that *Sphagneticola* × *guangdongensis* and the native species produced adventitious roots in every plant after 3 days under flooding stress, while the number of plants of the invasive species that produced adventitious roots reached its maximum after 4 days of flooding stress, at only about 20% (Figure 3c). Compared to the group not under flooding, flooding stress significantly reduced the number of stem nodes (Kruskal–Wallis test, F = 46.99, df = 5, *p* < 0.05; Mann–Whitney post hoc test), with the largest decrease observed in the invasive species (Figure 3d). Under flooding stress, the number of stem nodes in *S. trilobata* was lower than that in *Sphagneticola* × *guangdongensis* (*p* = 0.111) and *S. calendulacea* (*p* = 0.054). There was no significant difference in the number of stem nodes between *Sphagneticola* × *guangdongensis* and *S. calendulacea* (*p* = 0.739). However, without flooding stress, the number of stem nodes was the highest in the invasive species *S. trilobata,* compared to that in *Sphagneticola* × *guangdongensis* (*p* = 0.422) and the native species (*p* = 0.258). However, the stem node lengths of *Sphagneticola* × *guangdongensis* and the native species significantly increased under flooding stress (Kruskal–Wallis test, F = 53.16, df = 5, *p* < 0.05; Mann–Whitney post hoc test), and there was no significant change in the stem node length of the invasive species (Figure 3e) (*p* = 0.969).

### 2.4. Expression Levels of Ethylene Synthesis and Transduction-Related Genes

**Figure 4:**
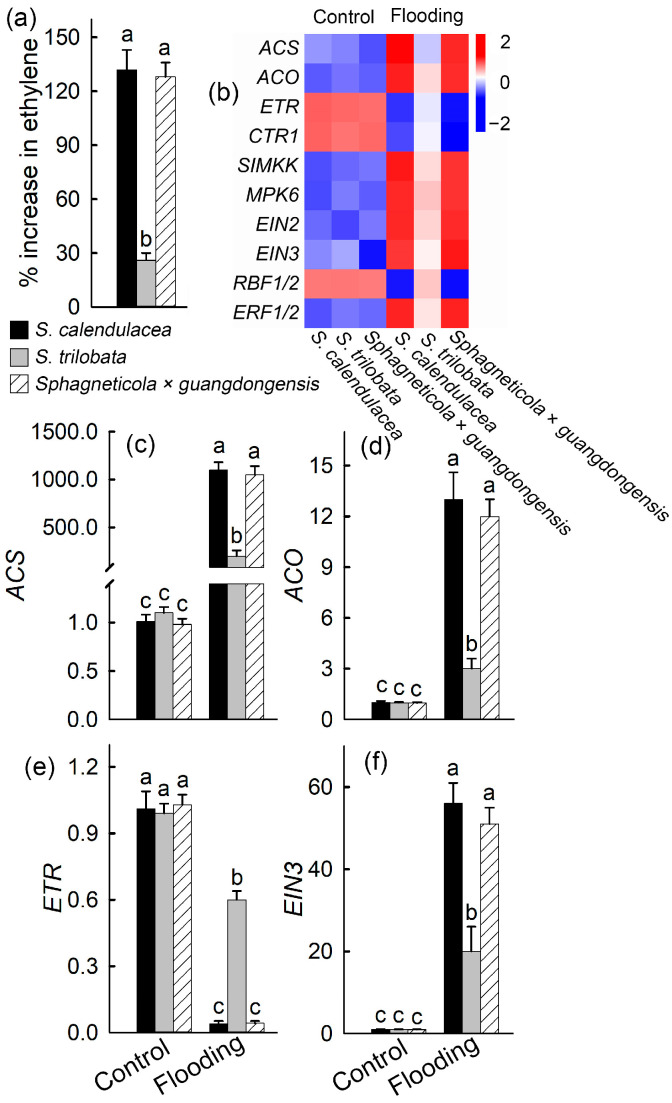
Percentage increase in ethylene content under flooding stress (**a**). Heat map of the genes related to ethylene synthesis and transduction (**b**) and expression of the selected genes were validated by real-time PCR under flooding stress and in the control group (**c**–**f**). There were three biological replicates. Different letters (a, b, c) above bars indicate significant differences (*p* < 0.05).

After flooding, the ethylene contents of the hybrid species *Sphagneticola* × *guangdongensis* (*p* < 0.01) and the native species *S. calendulacea* (*p* < 0.01) increased significantly, compared to the invasive species *S. trilobata*, but there was no significant difference between *Sphagneticola* × *guangdongensis* and the native species (Figure 4a) (F = 149.98; df = 2, 6; *p* = 0.644). Transcriptome analysis was conducted to investigate the expression of genes involved in ethylene synthesis and the signal transduction pathways under waterlogging stress. The expression levels of key enzyme genes for ethylene synthesis and positive regulatory genes in the ethylene signaling pathway were upregulated, with the expression levels of *Sphagneticola* × *guangdongensis* and the native species higher than those of the invasive species. The expression levels of negative regulatory genes in the ethylene signaling pathway were downregulated, with the expression levels of *Sphagneticola* × *guangdongensis* and the native species significantly lower than those of the invasive species (Figure 4b). The aminocyclopropane-1-carboxylic acid synthase gene (*ACS*) and aminocyclopropane-1-carboxylic acid oxidase gene (*ACO*) are the key enzymatic genes in ethylene biosynthesis, and the changes in their expression levels were consistent with the changes in the ethylene content. The ethylene response gene (*ETR*) and the ethylene insensitive3 gene (*EIN3)* are key enzyme genes for negative and positive regulation in ethylene signal transduction, respectively. The relative expression levels of *ACS*, *ACO*, *ETR,* and *NIE3* genes detected using real-time PCR were consistent with the transcriptome results (Figure 4c–f).

## 3. Discussion

The tolerance of the hybrid species *Sphagneticola* × *guangdongensis* and the native species *S. calendulacea* to flooding stress was stronger that of the invasive species *S. trilobata*. Several authors have reported that hybridization during plant invasion may produce more adaptable hybrid species [4,5,36]. For example, the hybridization between the native species *Spartina foliosa* and the invasive species *S. densiflora* enhanced the adaptability of the hybrid species to salt and flooding stress [7]. In our previous research, the tolerance of *Sphagneticola* × *guangdongensis* to cadmium and drought stress was inconsistent with the tolerance of *Sphagneticola* × *guangdongensis* to flooding stress in this study [32,33,34,35]. Ni et al. [31] found that the hybrid species *Sphagneticola* × *guangdongensis* was more sensitive to nitrogen deposition than the invasive species. The tolerance of *Sphagneticola* × *guangdongensis* to low temperature and low light was lower than that of the invasive species *S. trilobata* and the native species *S. calendulacea* [37]. The results of this study indicate that the hybrid species *Sphagneticola* × *guangdongensis* had stronger resistance to flooding stress than the invasive species *S. trilobata* and was closer to the native species *S. calendulacea*. As the flooding time continued, the leaves of both *Sphagneticola* × *guangdongensis* and its parents showed varying degrees of yellowing and shedding, with the invasive species suffering the most severe damage. After 2 days of flooding, necrotic spots appeared on the young leaves of the invasive species (Figure 1f). This implies that the tolerance of the invasive species was the weakest to flooding stress, while *Sphagneticola* × *guangdongensis* and the native species had stronger resistance to flooding stress. Flooding stress led to plant leaf decay and shedding, which was confirmed in other studies, and the species with stronger resistance suffered less damage on their leaves [38].

The net photosynthetic rate of the invasive species *S. trilobata* decreased the fastest, that of the native species *S. calendulacea* decreased the slowest, and that of the hybrid species *Sphagneticola* × *guangdongensis* decreased at a rate between its parents under flooding stress. Photosynthesis is the foundation of plant growth, and its changes under flooding stress reflect the degree of damage to plants [33]. Under flooding stress, the net photosynthetic rate of *Sphagneticola* × *guangdongensis* and its parents gradually decreased (Figure 2i), indicating that flooding stress caused damage to the plants [39]. Among them, the invasive species had the fastest decline rate, while *Sphagneticola* × *guangdongensis* and the native species had higher net photosynthetic rates than the invasive species, indicating that the invasive species suffered the most severe damage. Chlorophyll and Rubisco are the main substances responsible for light energy absorption and carbon fixation in photosynthesis, and changes in the chlorophyll and Rubisco can directly affect the net photosynthetic rate [40]. Flooding stress led to the degradation of chlorophyll and Rubisco in the plant leaves, resulting in a decrease in their contents. In this experiment, the contents of chlorophyll and Rubisco large subunits significantly decreased after flooding stress, with the highest contents being in the native species and the lowest being in the invasive species (Figure 2j,k). Previous studies also found decreases in the chlorophyll and Rubisco contents of *Arabidopsis* and *Prunus* leaves under flooding stress [13,28].

The invasive species *S. trilobata* suffered the most serious oxidative damage from flooding stress, compared to the hybrid species *Sphagneticola* × *guangdongensis* and the native species *S. calendulacea*. When the plants were subjected to flooding stress, the accompanying oxidative stress led to an increase in the content of ROS, and the accumulation of a large amount of ROS is toxic to plants [13,28,41]. In this study, the invasive species accumulated more H_2_O_2_ and O_2_^–^ than *Sphagneticola* × *guangdongensis* and the native species (Figure 2c,d). As toxic substances, H_2_O_2_ and O_2_^–^ can cause damage to plants by attacking their cell membranes and disrupting their biomolecules, among other means [41]. The degree of damage to membrane lipids can be indirectly reflected by the MDA content, which is the product of membrane lipid peroxidation. In this study, the MDA content in the leaves of the hybrid species *Sphagneticola* × *guangdongensis* and the native species *S. calendulacea* under flooding was lower than that of the invasive species, indicating that the tolerance of the invasive species was the weakest to flooding stress. Cell membrane damage led to higher ion leakage [42]. Under flooding stress, the cell membrane electron permeability of the invasive species was significantly higher than that of *Sphagneticola* × *guangdongensis* and the native species, but there was no significant difference between *Sphagneticola* × *guangdongensis* and the native species (Figure 2f). The tolerance of plants under flooding stress is related to the stability of their membrane structure. The MDA content and ion leakage are lower in plants with a strong tolerance [42,43,44]. These results imply that *Sphagneticola* × *guangdongensis* and the native species have greater tolerance to flooding stress than the invasive species.

The adaptive strategies of the hybrid species *Sphagneticola* × *guangdongensis* and the native species *S. calendulacea* under flooding stress were more effective than those of the invasive species *S. trilobata*. One of the critical strategies for plants to adapt to flooding stress is the formation of adventitious roots. The replacement of the malfunctioning primary roots by adventitious roots increases the plant’s access to oxygen and nutrients and enhances its tolerance to flooding stress [45]. The results showed that under flooding stress, *Sphagneticola* × *guangdongensis* and the native species formed a large number of adventitious roots quickly, while nearly 80% of the invasive plants did not form adventitious roots (Figure 3c). Aerenchyma is another important strategy for adapting to flooding stress, and its formation connects plants and transport oxygen obtained from the upper part downwards. Furthermore, it reduces the number of cells and the amount of oxygen consumption. After flooding, *Sphagneticola* × *guangdongensis* and the native species formed more aerenchyma than the invasive species (Figure 3b), which improves plant resistance under flooding stress [40]. These results suggest that the resistance of *Sphagneticola* × *guangdongensis* and the native species to flooding stress is stronger than that of the invasive species.

The formation of aerenchyma and adventitious roots was significantly positively correlated with changes in the ethylene content. Ethylene hinders the downward transport of auxin, promoting the formation of adventitious roots [46], and it increases cellulase activity and promotes the formation of aerenchyma [27]. The results showed that the increase in the ethylene content in *Sphagneticola* × *guangdongensis* and the native species was significantly higher than that in the invasive species after flooding stress (Figure 4a). Through transcriptome sequencing and real-time quantitative PCR analysis, it was found that the relative expression levels of the key enzyme genes *ASC* and *ACO* were significantly upregulated, which was positively correlated with ethylene synthesis [47,48], and the relative expression levels of *Sphagneticola* × *guangdongensis* and the native species were significantly higher than those of the invasive species. These genes, which are positively regulated in ethylene signal transduction, were upregulated, and their trends were consistent with those of the key enzyme genes in ethylene synthesis. The expression levels of the negative regulatory genes were downregulated, and the downregulation amplitude of the invasive species was the smallest (Figure 4b–f). The changes in gene expression were consistent with the changes in the ethylene content.

## 4. Materials and Methods

### 4.1. Plant Materials

The plant materials of *S. trilobata*, *S. calendulacea*, and their hybrid species *Sphagneticola* × *guangdongensis* were collected from the South China Botanical Garden, which is affiliated with the Chinese Academy of Sciences in Guangzhou, China [29]. These plant materials underwent a process of asexual propagation for cultivation. The stem with removed leaves was cut into about 10 cm and contained two pairs of buds for each piece. Subsequently, we placed the segments in an opaque container containing tap water and waited for them to take root and grow new leaves. Then, we transplanted the seedlings into growth substrate (a mixture of red soil and sand at a 3:1 ratio, *v*/*v*) in plastic pots. The seedlings exhibiting consistent growth after 28 days were chosen for the experiment. These selected seedlings were then divided into two groups: one group was subjected to flooding, while the other served as a non-flooding control. The plants in the flooding treatment group were placed individually into separate 100 L plastic containers filled with tap water. The plants in the flooding treatment were completely submerged in the tap water, with the water surface positioned 5 cm above the top of the plants. In contrast, the control treatment involved non-submerged plants that were irrigated daily. Five replications of each experimental unit, with 10 plants per treatment, were set. Samples with more than 5 biological replicates were taken from five experimental units separately, while samples with fewer than 5 biological replicates were randomly selected from five experimental units randomly.

### 4.2. Net Photosynthetic Rate and Chlorophyll Fluorescence Parameters

The Li-6800 photosynthesis measuring instrument (Li-Cor, Lincoln, NE, USA) was used to measure the leaf photosynthesis rate (Pn) using the method reported by Zhang et al. [49]. We selected one plant for each experimental unit, totaling five plants.

The chlorophyll fluorescence parameters were measured using a chlorophyll fluorescence imaging system (Technologica, Colchester, UK). Plant leaves were adapted at 900 μmol m^–2^ s^–1^ for 5 min, and then we determined the maximum fluorescence (Fm′) and steady fluorescence (Fs). The maximum fluorescence (Fm) and minimum fluorescence (Fo) were recorded after 30 min of dark adaptation under a 6000 μmol m^–2^ s^–1^ saturating pulse. The value of the effective quantum yield of the PSII (Φ_PSII_), the electron transfer rate (ETR), and the maximum photochemical efficiency (Fv/Fm) were calculated according to the methods in [50,51,52]. We selected one plant for each experimental unit, totaling five plants.

### 4.3. Rubisco Protein Western Blotting

Rubisco protein determination was performed using the method reported by Zhang et al. [49], with Bio-Rad 12% precast gradient gels. The Rubisco protein was detected with the anti-Rubisco antibody (Bioss, Beijing, China). Antibodies were diluted at 1:1000 in 5% non-fat milk in TBS-T.

### 4.4. Measurement of Electrolyte Leakage Rates and Malondialdehyde

A method described previously [53] was used to measure the electrolyte leakage rates. The FG3-ELK conductivity meter (Mettler Toledo, Zurich, Switzerland) was used to test the conductivity of the solution. The percentage of initial conductivity to the total conductivity was used to represent the rate of ion leakage. The malondialdehyde (MDA) content was measured according to the method published by Sun et al. [37]. We selected one plant for each experimental unit, totaling five plants.

### 4.5. Histochemical Staining

Nitro blue tetrazolium (NBT) histochemical staining was used to determine the superoxide anions (O_2_^–^), and diaminobenzidine (DAB) histochemical staining was used to determine the hydrogen peroxide (H_2_O_2_) [54].

### 4.6. Anatomical Analysis

We washed the adventitious roots of the plants in tap water and then cut them into 0.5 cm pieces using a blade. We soaked the cut roots in a centrifuge tube containing formalin and sank the roots to the bottom of the centrifuge tube by vacuum pumping. Dehydration was carried out using different concentrations of ethanol. Technovit 7100 resin (Wehrheim, Germany) was used to infiltrate the fixed roots, and then the fixed roots were embedded. Afterwards, they were stained with safranin (1%) and recorded using a microscope (Olympus CX30, Tokyo, Japan) after the cross-sections of the roots were sectioned [39].

### 4.7. Ethylene Measurement

The ethylene was measured as previously described [55]. In summary, the plants grew in a transparent enclosed container under flooding stress for 2 days. A 100 mL gas sample was collected, and the ethylene content was detected using gas chromatography (Varian CP-3800, Agilent Corporation, Santa Clara, CA, USA). We selected 3 plants randomly from the five experimental units.

### 4.8. Transcriptome Sequencing and Gene Expression Analysis

The method reported by Zhang et al. [35] was used to analyze the gene expression. The method described by Livak and Schmittgen [56] was used to analyze the genes’ relative expression levels. We selected 3 plants randomly from the five experimental units. The *GAPDH* gene was used as an internal reference, and the primer pairs were 5′-GGCTCGACTCGGCATATTCT-3′ (forward) and 5′-CGGCTGCCTTTGGTCTATGT-3′ (reverse). The primer pairs for the ethylene synthesis-related gene aminocyclopropane-1-carboxylic acid synthase gene (*ACS*) were 5′-GAGTTCGGTTAGCCCGTAGG-3′ (forward) and 5′-CGTTGTGGTCTCGTCATCCA-3′ (reverse), and for aminocyclopropane-1-carboxylic acid oxidase (*ACO*), they were 5′-TAGAGAAGCTGGCAGAGGGT-3′ (forward) and 5′-TGGCACGTCAATCCACTTGT-3′ (reverse). The primer pairs for ethylene signal transduction-related gene ethylene response (*ETR*) were 5′-CGTCGTTTGGCATCTGGTTC-3′ (forward) and 5′-TTTCTGGAGTTTGGAGGGCA-3′ (reverse), and for the ethylene insensitive3 gene (*EIN3*), they were 5′-CCAAAGCAGGATGGGTCTGT-3′ (forward) and 5′-AGAGGGCATCCTTGACTCCT-3′ (reverse). RNA sequencing data are available in the Gene Expression Omnibus (GEO) database under accession number SUB13842716.

### 4.9. Statistical Analysis

SPSS Statistics 19.0 (IBM, Armonk, NY, USA) was used for the statistical analysis. The data were subjected to a normal distribution test, and for data that conformed to a normal distribution, one-way analysis of variance was used. Duncan’s post hoc test was used for pairwise comparison. For data that did not conform to a normal distribution, the Kruskal–Wallis method was used, and the Mann–Whitney post hoc test was used for pairwise comparison. The significance was set as *p* < 0.05. SigmaPlot 12.5 (Systat Software Inc., Richmond, VA, USA) was used to conduct linear regression analysis and to plot the data. The values were expressed as the means ± standard errors.

## 5. Conclusions

**Figure 5:**
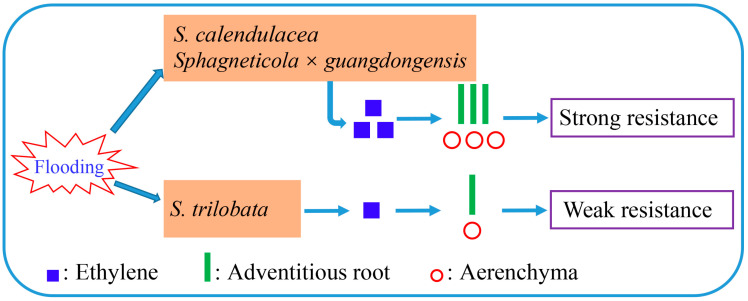
Schematic diagram of adaptation strategies to flooding stress in *Sphagneticola* × *guangdongensis* and its parents. Under flooding stress, the invasive species *S. trilobata* could not effectively synthesize and accumulate ethylene, resulting in a small number of adventitious roots and aerenchyma. However, the hybrid species *Sphagneticola* × *guangdongensis* and native species *S. calendulacea* synthesized and accumulated a large amount of ethylene rapidly, promoting the generation of adventitious roots and aerenchyma, which resulted in the resistance of *Sphagneticola* × *guangdongensis* and the native species being stronger than that of the invasive species.

This study indicated that hybridization with the native species *S. calendulacea* catalyzed the tolerance of the hybrid species *Sphagneticola* × *guangdongensis* to flooding stress. Under flooding stress, *Sphagneticola* × *guangdongensis* and the native species significantly upregulated the synthesis and accumulation of ethylene, promoted the formation of adventitious roots and aerenchyma, and improved their resistance to flooding stress, while the resistance in the invasive species was the weakest (Figure 5). Therefore, flooding stress may be a limiting factor for the spread and distribution of the invasive species *S. trilobata*, which may be overcome by hybridization with native species [57]. From a genetic perspective, this could be a method of gene invasion by spreading the genes of an invasive species through hybridization with a native species. Hybridization with native relatives is an important method for the successful invasion of invasive species.

## Data Availability

Data are contained within the article.
